# Supplementary material for: “It might be a statistic to me, but every death matters.”: An assessment of facility-level maternal and perinatal death surveillance and response systems in four sub-Saharan African countries
Source: PLoS One. 2020 Dec 18;15(12):e0243722. doi: 10.1371/journal.pone.0243722 (PMC7748147; doi:10.1371/journal.pone.0243722)
Supplement: S1 Table — (DOCX) [file pone.0243722.s001.docx]

## S1 Table. Data sources and collection methods

| **Data Source** | **Data Collection Method** |
| --- | --- |
| Subnational stakeholder interviews | - **Stakeholder semistructured questionnaires** included a 34-question questionnaire with questions on stakeholders’ specific role in supporting maternal and perinatal death surveillance and response (MPDSR) implementation, knowledge of current MPDSR practices, community linkages, and changes in care resulting from MPDSR processes. - Stakeholders at the district level were asked 12 additional, more detailed questions on local knowledge of and experience supporting implementation of MPDSR, including aggregation and use of surveillance data at district level, and risks and benefits associated with the MPDSR process. |
| Facility visits to assess MPDSR practice and interviews with staff at facilities | - **Facility questionnaires** were used in semistructured, in-person interviews with facility health workers involved in MPDSR activities. The questionnaire includes 66 questions addressing a range of activities in the MPDSR continuous action cycle and respondent perceptions of MPDSR processes, including a no-blame culture, sources of support (e.g., training, supervision, funding), and incentives. - **Reviews** of facility MPDSR documents substantiate facility respondent descriptions of key facility MPDSR activities and records. Examples of common documents reviewed include MPDSR forms (including examples of completed forms), facility registers, health management information system reports, and MPDSR meeting notes. All documentation collected contained no identifiers. |
