# Supplementary material for: “It might be a statistic to me, but every death matters.”: An assessment of facility-level maternal and perinatal death surveillance and response systems in four sub-Saharan African countries
Source: PLoS One. 2020 Dec 18;15(12):e0243722. doi: 10.1371/journal.pone.0243722 (PMC7748147; doi:10.1371/journal.pone.0243722)
Supplement: S2 Table — (DOCX) [file pone.0243722.s002.docx]

## S2 Table. Mapping content of national MPDSR policy by country

|  | **Nigeria** | **Rwanda** | **Tanzania** | **Zimbabwe** |
| --- | --- | --- | --- | --- |
| Name of policy | National Guidelines for Maternal and Perinatal Deaths Surveillance and Response in Nigeria (2015; adopted in 2016) | Maternal Death Surveillance and Response: Technical Guideline (January 2015) | Maternal and Perinatal Death Surveillance and Response (MPDSR) Guideline (2015) | Guidelines for Maternal and Perinatal Death Audits in Zimbabwe (2013) |
| Is the policy integrated (maternal and perinatal)? | Yes | No | Yes | Yes |
| Does the policy have clear links to a health management information system (HMIS) and/or a civil registration and vital statistics system? | The policy calls for links to the HMIS and integrated disease surveillance and response but does not provide clear guidance. | The policy indicates that deaths are recorded in the electronic integrated disease surveillance and response instance of the national HMIS. | Yes, key indicators are included.  At the national level, the Ministry of Health, Community Development, Gender, Elderly, and Children reported that it is working on strengthening MPDSR systems by reviewing the current MPDSR-HMIS integration with plans to upload documentation and notification forms in the near future. | Key indicators are included in the HMIS. |
| Does the policy have tools? If so, include the names of the tools provided. | - Maternal death notification form - Health facility-based maternal death review form - Recommendations and action plan form - Quarterly summary report form for response tracking - Maternal death review for identification number coding instructions - Perinatal death notification form - Health facility-based perinatal death review form   Community-level versions of all forms exist, where verbal autopsy is performed. | - Notification and identification form for maternal death in the community - Notification and identification form for maternal death in the facility - Facility-based maternal death review reporting form   Neonatal deaths and stillborn audits have standard forms. | - Maternal death review form - Perinatal death review form - Action plan example | - Maternal death notification form - Maternal death preliminary assessment form - Maternal death assessment form - Monthly statistics data sheet - Perinatal death notification form - Stillbirth and early neonatal death assessment form |
| Are the tools electronically available? | Not at time of assessment, but two types of electronic tools were introduced in May 2017 and are currently being field-tested in nine of 36 states, preparatory to their harmonisation. | Yes, for perinatal and maternal, but the maternal system is not used. Facilities complete an Excel file and submit this to the Ministry of Health. | - Not at the time of the assessment, though MPDSR electronic tools were in place and ready to be disseminated in 2018. | Electronic tools are being piloted in two provinces. |
| What method does the policy and/or tools use to classify cause of death? | The policy includes the following guidance: “classification of the type and primary causes of maternal and perinatal death.” Although the policy and tools demand coding using the 10th edition of the *International Statistical Classification of Diseases and Related Health Problems* (ICD-10), it was observed at trainings that capacity for ICD coding for maternal and perinatal mortality was low across the country. | Facilities report using the standard checklists provided in the notification forms. The methods include:   - By direct obstetric cause - By mode of delay - By preventability | ICD-10 coding is proposed in guideline.  Death registers for the HMIS use different coding from ICD-10 because of lack of training on ICD-10 coding. | The maternal and perinatal death assessment forms use the following classifications in the conclusion section:   - Direct/indirect/incidental - Three delays - Avoidable/possibly avoidable/not avoidable |
| Does the policy have clear implementation guidance? | Yes, multiple schematics show how the system should function, including rollout and reporting. | Yes, for maternal death audit. The guideline has clear algorithms to guide implementation.  However, there is no guideline for perinatal death audit. | Yes, clear guidance is provided on how to implement, including how to set up committees, conduct meetings, and report notification and review forms. Guidance on how to follow up on action plan could be strengthened. | Yes, very clear guidance is provided on step-by-step implementation of all stages of the MPDSR cycle except the response component, which requires strengthening. |
| Are there recommendations linked to the revisions of the guidelines or policy? | No | Yes, there is an expressed need to revise and update guidelines by integrating maternal and perinatal death audits. | Yes, there is a plan to review MPDSR guidelines to update data according to Tanzania Demographic Health Survey 2015/2016 data and inclusion of ICD-10 for perinatal mortality, which is missing. | Yes, review the existing guideline with the following main goals:   - The Ministry of Health and Child Care and the MPDSR national steering committee develop a formal system along with tools for tracking and monitoring MPDSR implementation as per the guideline, going beyond just the action plan framework. Facilities, county health management teams, and regional health management teams should be provided with tools to help them track and evaluate the response portion of the implementation process. - Review the existing coding system, align it as per World Health Organization guidelines (e.g., ICD maternal and perinatal mortality), and ensure it is captured in registers and notification forms used in health facilities. |
