# Supplementary material for: “It might be a statistic to me, but every death matters.”: An assessment of facility-level maternal and perinatal death surveillance and response systems in four sub-Saharan African countries
Source: PLoS One. 2020 Dec 18;15(12):e0243722. doi: 10.1371/journal.pone.0243722 (PMC7748147; doi:10.1371/journal.pone.0243722)
Supplement: S3 Table — (DOCX) [file pone.0243722.s003.docx]

## S3 Table. Summary of MPDSR implementation enablers and barriers most commonly cited by facility staff in four countries

|  | Nigeria* | Rwanda | Tanzania | Zimbabwe |
| --- | --- | --- | --- | --- |
| Main factors that facilitated implementation of MPDSR | - Staff commitment - Community involvement - Evidence of MPDSR process improving health services | - Facility leadership - Teamwork, communication among staff, staff commitment, and participation during meetings - Support from national/subnational managers, including MPDSR training support | - Teamwork, communication among staff, staff commitment, and participation during meetings - Availability of MPDSR guideline and tools - Evidence of MPDSR process improving health services - Support from national/subnational level, including with capacity-building through trainings - No-blame environment | - Teamwork, communication among staff, staff commitment, and participation during meetings - Staff motivation triggered by observed increase in deaths - Support from national/subnational level, including with capacity-building through trainings - Availability of the guideline and tools - Evidence of process improving health services |
| Barriers to MPDSR implementation | - Guidelines and forms not available - Limited staff time and work overload preventing staff from attending meetings | - Limited staff time and work overload preventing staff from attending meetings - Human resource shortage including high staff turnover and staff shortage - Lack of leadership either from subnational/national level or facility level - Limited staff motivation | - Limited staff time and work overload preventing staff from attending meetings - Human resource shortage, including high staff turnover and staff shortage - Lack of training - Challenges in collecting information about the cases for proper analysis - Limited staff motivation - Demotivating, specifically because recommendations are not implemented | - Limited staff time and work overload preventing staff from attending meetings - Blame environment/poor staff attitudes - Limited staff motivation |
| Most helpful activities to improve facility MPDSR processes | - Increase staff numbers. - Improve response to implementation of action plans with additional resources. | - Provide motivation and/or incentives for staff involved in MPDSR. - Offer additional capacity-building and training for staff. - Strengthen facility leadership. - Increase hiring and retention of qualified staff. - Strengthen response/action to proposed action plans developed through audit process. | - Offer additional capacity-building and training for staff. - Provide motivation and/or incentives for staff involved in MPDSR. - Increase hiring and retention of qualified staff. - Improve review meetings (e.g., broader participation, regularity, and blame-free environment). - Increase funding to support MPDSR process, including meetings and follow-up to response. - Strengthen response/action to proposed action plans developed through audit process. - Strengthen facility leadership. | - Increase funding to support MPDSR process, including meetings and follow-up to response. - Provide and improve tools and reporting methods (e.g., digitise system). - Offer additional capacity-building and training of staff. - Provide motivation and/or incentives for staff involved in MPDSR. - Improve review meetings (e.g., broader participation, regularity, and blame-free environment). - Strengthen response/action to proposed action plans developed through audit process. - Strengthen facility leadership. |

Source: Common themes identified based on open-ended responses of facility interviews. Themes listed in order of frequency reported by facility staff in each country; responses only included if mentioned by more than one facility in each country.

*The assessment included responses from two of the three health facilities in Nigeria that responded to these questions.
