# Supplementary material for: “It might be a statistic to me, but every death matters.”: An assessment of facility-level maternal and perinatal death surveillance and response systems in four sub-Saharan African countries
Source: PLoS One. 2020 Dec 18;15(12):e0243722. doi: 10.1371/journal.pone.0243722 (PMC7748147; doi:10.1371/journal.pone.0243722)
Supplement: S4 Table — (DOCX) [file pone.0243722.s004.docx]

## S4 Table. Ranking of progress markers by frequency across 47 facilities

| Progress Marker | Instrument Items | Number of Points Allocated (by facility to progress marker) | Proportion of Points Allocated to the 47 Facilities |  |
| --- | --- | --- | --- | --- |
| Number and type of (senior) managers involved in implementation process (in relation to size of facility) | Special person(s) who take specific effort in promoting death reviews, including management, professionals, driving forces (contact person, meeting coordinator, other champion) | *1 point* | 98% |  |
| Decision to implement MPDSR | Knowledge of the original decision to implement death reviews. If death reviews have not yet been implemented, has a formal decision been made? | *1 point* | 94% |  |
| Meeting process established | Ability to describe or show documentation of meeting process | *0.5 points* | 94% |  |
| Steering committee | A death review leadership team or steering committee is established. | *1 point* | 91% |  |
| Number and type of (senior) managers involved in implementation process (in relation to size of facility) | Clear leader(s) are involved in establishing and championing death reviews (past or future). | *1 point* | 89% |  |
| Multidisciplinary meetings | Death review meetings include staff from different disciplines and management. | *2 points* | 88% |  |
| Documented results | Facility records show ongoing death review meetings for at least 1 year. | *2 points* | 85% |  |
| Tools available | A data collection form is available. | *1 point* | 84% |  |
| Tools available | Tools include modifiable factors. | *1 point* | 84% |  |
| Tools available | Tools include cause of death. | *1 point* | 83% |  |
| Evidence of MPDSR meetings | Meeting minutes are available. | *1 point* | 76% |  |
| Orientation for new staff | Face-to-face or written orientation on death reviews is available for new staff. | *1 point* | 72% |  |
| Evidence of MPDSR meetings | Meeting minutes include action items. | *1 point* | 69% |  |
| Evidence of MPDSR meetings | Meeting notes respect confidentiality of staff and patients. | *1 point* | 69% |  |
| Score on the first five stages (divided by 12) |  | *2 points* | 64% |  |
| Evidence of routine MPDSR practice | Death review meetings are held at stated interval (e.g., weekly, monthly). | *1 point* | 54% |  |
| Tools available | Tools include place to follow up on actions taken. | *1 point* | 49% |  |
| Evidence of staff development | There is evidence that staff have received MPDSR training in the past year. | *1 point* | 46% |  |
| Further evidence of practice | There is evidence of change based on recommendations that arise from death review findings. | *3 points* | 45% |  |
| Community linkages | There is evidence of reporting findings and progress to the community. | *1 point* | 35% |  |
| MPDSR data use | Data trends are displayed or shared. | *2 points* | 34% |  |
| Resources allocated | Allocations from the hospital budget or support from other partners to establish death reviews | *1 point* | 32% |  |
| Evidence of MPDSR meetings | Meeting minutes include follow-up from previous meetings. | *1 point* | 31% |  |
| Evidence of staff development | There is a plan in place to ensure all staff receive MPDSR training. | *1 point* | 24% |  |
| Meeting process established | A staff meeting conduct agreement is available. | *0.5 points* | 9% |  |
|  |  | *30 points* |  |  |
