# Supplementary material for: “It might be a statistic to me, but every death matters.”: An assessment of facility-level maternal and perinatal death surveillance and response systems in four sub-Saharan African countries
Source: PLoS One. 2020 Dec 18;15(12):e0243722. doi: 10.1371/journal.pone.0243722 (PMC7748147; doi:10.1371/journal.pone.0243722)
Supplement: S1 File — (DOCX) [file pone.0243722.s005.docx]

## S1 File. Brief historical summary of MPDSR processes by country

**Nigeria**

For many years in Nigeria, no harmonised or standard process for reviewing maternal and perinatal deaths existed; implementation depended on individual health facilities conducting clinical audits, mainly pertaining to maternal deaths. With the release of World Health Organization (WHO) guidelines for maternal death surveillance and response (MDSR) in 2013 and growing national interest, the Federal Ministry of Health (FMOH) sought to operationalise a mortality audit system for the reduction of maternal and perinatal deaths. The FMOH adopted MDSR in 2013 and an integrated maternal and perinatal death surveillance and response (MPDSR) guideline in 2015, after advocacy for inclusion of stillbirths and neonatal deaths into the MDSR system. The National Council on Health approved the MPDSR guideline and tools in 2015.

The national guidelines, data collection registers, and other tools aim to achieve routine tracking and review of all maternal and perinatal deaths in Nigeria. The government has since disseminated this policy and directed state governments throughout the country to implement MPDSR. Currently, there is no routine tracking system for the rollout of MPDSR and no way to monitor those local government areas (LGAs) and facilities that have begun, ceased, or continue to implement any form of death review.

**Rwanda**

Maternal death audit (MDA) committees were established in Rwanda in 2008, and hospitals began conducting facility-based MDA in 2009[.](#_ENREF_5) MDA approaches included facility-based deaths audit, verbal autopsy, and confidential inquiry into maternal deaths. Standard tools for these three approaches were adapted to the local context, and health providers from all hospitals received training. Neonatal death and stillborn audits began in 2010 and 2015, respectively. At the time of the assessment, MDAs used standard audit forms with tools available depending on the approach used, whereas stillbirths and neonatal audits only used standard forms.

In 2014, after the release of the WHO MDSR technical guidance, Rwanda transitioned to MDSR, formed a national MDSR committee, published the National Technical Guideline for MDSR, distributed notification and review forms, and organised trainings for health providers (2015), including doctors, obstetricians, paediatrics, and midwives from teaching, referral, provincial, and district hospitals. District hospital providers trained staff from health centres. Rwanda is currently in the process of revising the national MDSR technical guidelines to incorporate neonatal deaths and stillbirths.

**Tanzania**

Since 1984, select health facilities in Tanzania have been conducting MDAs. In 2006, maternal and perinatal death reviews (MPDRs) became required, routine practice in Tanzanian health facilities after release of national MPDR guidelines. Due to weak scale-up of the MPDR, poor monitoring and evaluation, and inadequate expertise to analyse problems and lack of response and solutions, Tanzania began revising its national MPDSR guidelines in 2013 with an emphasis on the response. Building on the global momentum to implement MDSR systems, Tanzania released a national MPDSR guideline and tools in November 2015, providing orientation/training across all regions.

The national guideline and data collection registers and other tools aim to achieve routine tracking and review of all maternal and perinatal deaths in Tanzania. The Tanzanian government has disseminated this guideline to most health facilities and directed regional and LGAs to implement the audit process. Currently, there is no routine tracking system for the rollout of MPDSR and no way to monitor implementation progress and processes at LGA and facility levels.

**Zimbabwe**

The central hospitals in Zimbabwe had been conducting maternal and perinatal mortality audit meetings for over 30 years before the health system challenges in 2007. Facilities used these meetings to improve quality of care at facilities, but they were not coordinated nationally and did not systematically report deaths and track trends. After WHO released guidelines for maternal audits in 2004, the Ministry of Health and Child Care (MOHCC) instituted a requirement that notification forms be completed for every maternal death, in line with 2006 legislation. In 2013, the MOHCC developed guidelines for conducting maternal and perinatal death audits at health facilities. In 2016, the MOHCC minister appointed a national MPDSR committee to oversee and coordinate efforts to eliminate preventable maternal and perinatal mortality by obtaining and using facility mortality information to guide public health actions and monitor their impact. Provincial programme leaders received national-level training on the new guidelines, and all facilities received hard copies.
