# Supplementary material for: “It might be a statistic to me, but every death matters.”: An assessment of facility-level maternal and perinatal death surveillance and response systems in four sub-Saharan African countries
Source: PLoS One. 2020 Dec 18;15(12):e0243722. doi: 10.1371/journal.pone.0243722 (PMC7748147; doi:10.1371/journal.pone.0243722)
Supplement: S1 Data — (DOCX) [file pone.0243722.s006.docx]

## S1 Data: Database for MCSP Multicountry Assessment of MPDSR implementation

Available in the embedded excel file:
