# Supplementary material for: “It might be a statistic to me, but every death matters.”: An assessment of facility-level maternal and perinatal death surveillance and response systems in four sub-Saharan African countries
Source: PLoS One. 2020 Dec 18;15(12):e0243722. doi: 10.1371/journal.pone.0243722 (PMC7748147; doi:10.1371/journal.pone.0243722)
Supplement: Attachment — Submitted filename: Response to reviewers Nov 2020.docx [file pone.0243722.s008.docx]

*“It might be a statistic to me, but every death matters.”:* An assessment of facility-level maternal and perinatal death surveillance and response systems in four sub-Saharan African countries

# Response to editors comments

| Journal requirements | Response/action |
| --- | --- |
| Thank you for responding to the reviewers' comments. | Thank you for sharing the revised manuscript with the reviewers for additional comment. We are pleased to see that the peer reviewers had no further comments beyond minor editorial issues.  We sincerely hope that the changes made on this second revision will suffice for acceptance for the editors. The paper was originally submitted on January 7, 2020. The peer review comments have been overall positive and constructive, and our revisions submitted within the timeframe allocated by the journal. We completely understand that COVID-19 has made academic work extremely challenging and multiple editors have been assigned to this paper. Nonetheless, we hope the journal can work swiftly to make a decision about the paper upon receipt of this second revision. The research presented in this paper informs implementation of an intervention (MPDSR) in low- and middle-income settings, and we are eager to see this work published in order to support future implementation processes. |
| It appears that you edited the reference list; however, there are no tracked changes. Please indicate those edits with tracked changes. | Thank you for noting that there were no track changes on the reference list. For the revised manuscript, we used a referencing software to update the references in order to minimize human error when addressing the corrections. Unfortunately, the system does not allow for track changes. For this revised resubmitted version, we have removed the software from the manuscript and have made the previous edits with track change. |
| You noted that you based the tool on literature, expert opinion, and national policies; however, Table 3 indicates justifications for tool elements based only on literature. | Thank you for your comment. We have made edits to clarify the policy mapping process and how this links to the results and discussion. As described, the tool itself was conceptually adapted from a tool used to measure Kangaroo Mother Care implementation. We used the MPDSR literature, expert opinion and the existing global guidelines for perinatal death audit and MDSR to inform the content. We had amended the sentence to remove national guidelines as a source to inform the tool content. References to the global guidelines are included among the references in Table 3. |
| You mention policy differences at different points in the Discussion--around lines 410-420 and 475--but the reader doesn't get a sense of what the national policies contain, how they differ from each other, and how they differ from global standards in the main text. The information in Supplemental Files 1 and 2 is useful but we still can't see how the policies measure up against the tool. In other words, is the tool measuring things not contained in the policies for one or more countries? And might national policy deviance from literature or global standards account in part for the scoring? It would be useful to know if facility scores result more from a lack of adequate national policy or lack of capacity at facilities. You seem to imply the latter without addressing the former possibility. This issue would benefit from an organized discussion rather than mentioning policy issues in different places. | The findings of the policy mapping are presented at the start of the Results section (lines 172-180) with details in Supplemental file 2 (Table S2.1). It is helpful to have your perspective regarding the possible confusion around the links of the tool and the policy analysis, and we appreciate you flagging this concern. We have made edits to the discussion section to address your questions. |

# Response to reviewers' comments

| **Comments to the Author** | **Response from authors** |
| --- | --- |
| REVIEWER #1 |  |
| just a few typos in the revised version, e.g. "characterstics" instead of "characteristics", line 543, p.28. | Thank you. We have made this correction and have done another review to check for other typos. |
| Also, please, standardize the references so that we don't have sometimes the full name of the journal and sometimes a short name. | Thank you for noting the issues in the references. The journal’s referencing style, Vancouver, requires the titles of journals to be abbreviated as they appear in the Journals in NCBI Databases. <https://www.ncbi.nlm.nih.gov/nlmcatalog/journals> We have gone through each reference and corrected accordingly. |
| REVIEWER #2 |  |
| No comments | Thank you. |
